# A Web-Based Knowledge Translation Resource for Families and Service Providers (The “F-Words” in Childhood Disability Knowledge Hub): Developmental and Pilot Evaluation Study

**DOI:** 10.2196/10439

**Published:** 2018-12-21

**Authors:** Andrea Cross, Peter Rosenbaum, Danijela Grahovac, Julie Brocklehurst, Diane Kay, Sue Baptiste, Jan Willem Gorter

**Affiliations:** 1 CanChild Centre for Childhood Disability Research McMaster University Hamilton, ON Canada; 2 School of Rehabilitation Science Faculty of Health Sciences McMaster University Hamilton, ON Canada; 3 Department of Pediatrics McMaster University Hamilton, ON Canada

**Keywords:** childhood disability, F-words, ICF, Knowledge Hub, knowledge-to-action framework, knowledge translation, Web-based resource

## Abstract

**Background:**

The “F-words in Childhood Disability” (Function, Family, Fitness, Fun, Friends, and Future) are an adaptation and an attempt to operationalize the World Health Organization’s (2001) International Classification of Functioning, Disability and Health (ICF) framework. Since the paper was published (November 2011), the “F-words” have attracted global attention (>12,000 downloads, January 2018). Internationally, people have adopted the “F-words” ideas, and many families and service providers have expressed a need for more information, tools, and resources on the “F-words”.

**Objective:**

This paper reports on the development and pilot evaluation of a Web-based knowledge translation (KT) resource, the “F-words” Knowledge Hub that was created to inform people about the “F-words” and to provide action-oriented tools to support the use of the “F-words” in practice.

**Methods:**

An integrated research team of families and researchers at *CanChild* Centre for Childhood Disability Research collaborated to develop, implement, and evaluate the Knowledge Hub. A pilot study design was chosen to assess the usability and utility of the Web-based hub before implementing a larger evaluation study. Data were collected using a brief anonymous Web-based survey that included both closed-ended and open-ended questions, with the closed-ended responses being based on a five-point Likert-type scale. We used descriptive statistics and a summary of key themes to report findings.

**Results:**

From August to November 2017, the Knowledge Hub received >6,800 unique visitors. In 1 month (November 2017), 87 people completed the survey, of whom 63 completed the full survey and 24 completed 1 or 2 sections. The respondents included 42 clinicians and 30 family members or individuals with a disability. The majority of people visited the Knowledge Hub 1-5 times (n=63) and spent up to 45 minutes exploring (n=61) before providing feedback. Overall, 66 people provided information on the perceived usefulness of the Knowledge Hub, of which 92% (61/66) found the Knowledge Hub user-friendly and stated that they enjoyed exploring the hub, and a majority (n=52) reported that the Knowledge Hub would influence what they did when working with others. From the open-ended responses (n=48), the “F-words” videos (n=21) and the “F-words” tools (n=15) were rated as the best features on the Knowledge Hub.

**Conclusions:**

The “F-words” Knowledge Hub is an evidence-informed Web-based KT resource that was useful for respondents, most of whom were seen as “early adopters” of the “F-words” concepts. Based on the findings, minor changes are to be made to improve the Knowledge Hub before completing a larger evaluation study on the impact at the family, clinician, and organizational levels with a wider group of users. Our hope is that the “F-words” Knowledge Hub will become a go-to resource for knowledge sharing and exchange for families and service providers.

## Introduction

### Background

It has been several years since the paper *“The ‘F-words’ in Childhood Disability: I swear this is how we should think!”* was published in Child: Care, Health and Development [1]. The “F-words” (Function, Family, Fitness, Fun, Friends, and Future) are an adaptation and operationalization of the World Health Organization’s International Classification of Functioning, Disability and Health (ICF) [2]. The initial aim of the “F-words” paper was to spread awareness of the ICF and to encourage people to apply these modern ways of thinking and developmental approaches to childhood disability [1]. Since it was first published (early November 2011) to December 2017, the paper has been cited over 140 times and downloaded over 12,000 times.

In 2014, based on considerable interest in the paper, we formed an integrated research team at *CanChild* Centre for Childhood Disability Research focused on disseminating the “F-words” into practice. At that time, several parents (ie, *early adopters*) had learned about the “F-words,” liked the ideas, and were interested in how to share the “F-words” message with more families. Recognizing the potential impact of an integrated approach to this work (ie, families and researchers working together), we partnered with family stakeholders to develop and evaluate knowledge translation (KT) strategies tailored to meet the families’ needs and preferences.

The first project involved the development, dissemination, and evaluation of a three-minute awareness video [3]. A video was chosen as an initial dissemination strategy as it was engaging, relatively easy to produce, and could be freely shared with a broad audience. At that time, the “F-words” paper was not yet open access and thus was reaching a limited audience in the scientific and clinical communities.

We evaluated the video by tracking its reach and asking viewers to complete an anonymous Web-based survey. In the first 2 months, there were 715 views and 137 survey responses. Overall, we learned that 97.8% (134/137) of people “extremely liked” or “liked” the “F-words” ideas, 87.5% (120/137) indicated they would share the video, and 92.7% (127/137) wanted to learn more. The *CanChild* website was identified by 65.7% of respondents (90/137) as the most popular strategy for sharing further information on the “F-words” concepts. A complete report of our findings and the lessons learned from this project are published [3].

The awareness video was only the first step toward moving the “F-words” into practice. By January 2015, we had given >30 international presentations and the “F-words” ideas had continued to spread over social media. We were gratified by the uptake of these ideas around the world and were excited to see the imaginative ways in which people were adapting and adopting the “F-words” to local contexts. We were also learning a great deal about the application of the “F-words” by connecting and working with families and other stakeholders such as service providers and health care administrators around the world. Therefore, as a research team, we were acting as “knowledge brokers” [4] by working with interested people to share and exchange knowledge on the “F-words” concepts.

From our conversations with the families and service providers, it was evident that there was significant interest in having more information on the “F-words” as well as action-oriented resources and tools to assist with the application of the “F-words” into practice. Furthermore, as the “F-words” ideas continued to spread, we recognized the need (and opportunity) to compile and share all that was being done on the “F-words” ideas by building a centralized Web-based community for knowledge sharing and exchange. Therefore, in 2015, our research team decided to develop, implement, and evaluate the usability and utility of a Web-based KT resource: a website called “The ‘F-words’ in Childhood Disability Knowledge Hub.”

The purpose of the “F-words” Knowledge Hub was to inform families and service providers about “F-words”/ICF concepts and to provide action-oriented tools to support the uptake and use of the “F-words” in practice. The Knowledge Hub is currently hosted on *CanChild’s* website [5] and is meant to be an ever-growing resource for knowledge sharing and exchange. The *CanChild* website is world-renowned in the field of childhood disability and receives over 12,000 unique visitors each month from over 205 countries [6].

### Modern Approaches to Knowledge Translation

In the last several years, there has been increasing interest in using the internet as a platform for KT and the use of Web-based KT resources as a strategy for disseminating health research evidence in the field of childhood disability [7-10]. Levac et al [7] defined Web-based KT resources as “e-learning products that translate evidence-based knowledge to disseminate information that increases awareness, informs clinical practice, and stimulates practice change.” The Web-based KT resources include websites, educational modules, downloadable PDFs, blogs, and wikis [7,11]. Some of the advantages of Web-based resources are (1) the ability to be self-paced or self-directed; (2) accessibility and broad reach; (3) incorporation of engaging multimedia content; and (4) promotion of knowledge sharing and exchange [7,12].

While the current evidence base for Web-based KT strategies is limited, some studies have shown promising findings [12,13]; however, more research is needed to identify the most effective Web-based KT strategies and to understand their impact on behavior change and patient outcomes [13,14]. Research is also needed to explore the impact of Web-based KT resources as a single intervention compared with multifaceted interventions, such as a combination of Web-based KT resources and educational outreach [12,13].

This paper reports on the development process, usability, and utility of the Knowledge Hub. The Knowledge-to-Action (KTA) framework was used as the guiding theoretical underpinning for this research [15]. KT theories, models, and frameworks are recommended to guide the development, implementation, and evaluation of KT strategies [16-18]. The KTA framework provided a conceptual map of the KT process steps involved in transferring knowledge to practice [15]. For this study, we focused on the three steps of the *action cycle: “select, tailor, and implement the intervention,” “monitor knowledge use,”* and *“evaluate outcomes.”* This study was part of a larger research program that had already addressed the earlier stages of the *action cycle* [3].

## Methods

### Integrated Knowledge Translation Strategy

We implemented a formal integrated knowledge translation (iKT) strategy to develop, implement, and evaluate the Knowledge Hub. iKT involves the collaboration of researchers and knowledge users (eg, families and service providers) throughout all stages of the research or KT process [19] and has been found to increase the effectiveness and sustainability of KT interventions [20,21]. This project was led by an integrated team of children’s health researchers (PR, SB, JWG), family stakeholders (DG, JB, DK), and a doctoral student (AC), who coordinated the project.

All team members were involved in each stage of the project: (1) participating in initial planning stages; (2) providing feedback on the content and design of the Knowledge Hub; (3) creating and sharing tools/resources; (4) assisting with evaluation; and (5) disseminating the hub across their social networks. During the initial planning stages, team meetings were held by teleconference. We initially planned to develop an “F-words” Tool Kit as a paper-based resource designed to share knowledge and provide tools/resources to support the use of the “F-words” in practice; however, after extensive conversations with stakeholders and a review of the literature, we turned toward Web-based KT strategies (ie, the Knowledge Hub). AC led the development of the hub, but feedback was sought and received from all team members throughout the development process. Most team correspondence was done through email.

An area in which all three family stakeholders were heavily involved was the creation of the “F-words” tools; the “F-words” agreements, photo collage, goal sheet, and profile. Many of the ideas for the tools came from family stakeholders’ personal experiences of working with service providers and their perceptions of how the “F-words” could be used in practice. As an integrated research team, we discussed the purpose and goals for each tool, and then with the support of *CanChild* students we developed draft tool templates that could be distributed to all team members for feedback. When all team members had approved the tools, they were then posted on the Knowledge Hub.

### Knowledge Hub Development Process

To help with the planning and development of the Knowledge Hub, we used Levac et al’s [7] best practice recommendations for designing Web-based KT resources. These were based on their experiences developing and evaluating Web-based KT resources, as well as a review of the KT and instructional design literature [7]. They identified four main recommendations: (1) develop evidence-based user-centered content; (2) tailor content to the Web-based format; (3) evaluate impact; and (4) share the results and disseminate knowledge. Each recommendation had several specific steps; the full description of the application of Levac et al’s [7] recommendations for this study is provided in Multimedia Appendix 1.

### Description of Knowledge Hub

The purpose of the Knowledge Hub is to have a single site where people can go to learn about and share ideas for utilizing the “F-words” concepts in practice. The Web-based hub [22] includes tools and resources created by our research team, as well as materials that have been generously shared by stakeholders from around the world. Everything on the Knowledge Hub is freely available to share and adapt to localized practice settings. The Knowledge Hub has 6 main sections: (1) the F-words Homepage; (2) ICF Resources; (3) F-words Footprint; (4) Family & Clinician Voices; (5) F-words Tools; and (6) F-words Research Team. A full description of the Knowledge Hub is provided in Multimedia Appendix 2.

### Knowledge Hub Evaluation

A pilot study design was used to assess the usability and utility of the Knowledge Hub, and to make any necessary changes, before implementing a larger evaluation study. Usability was measured with “usefulness” questions (ie, clear purpose, user-friendly, content meaningful or relevant) and utility was measured using “use” questions (ie, impact and use intent, change in knowledge, attitude, and behavior). Usability and utility testing is a critical component to the success of KT interventions [7,23]. Visitors to the Knowledge Hub were asked to review the hub and voluntarily provide feedback by completing a brief anonymous Web-based open survey through McMaster University’s LimeSurvey system. Participants were told that by completing the survey they were giving their consent to participate in the study. A survey link was posted on the Knowledge Hub, and a recruitment email and poster were distributed through *CanChild’s* social networks. The recruitment poster is provided in Multimedia Appendix 3.

The survey included both closed-ended and open-ended questions. The closed-ended responses had a five-point Likert-type scale that evaluated the visitors’ prior familiarity with the “F-words”, the perceived usefulness, and reported or intended use of the Knowledge Hub. Adaptive questioning was used (ie, some questions were conditionally displayed based on the responses to previous questions) to reduce the complexity of the survey. There were 37 questions in the survey. Google analytics evaluated the reach by tracking the number of visitors to the hub over a four-month period. Descriptive statistics were used to analyze the quantitative information, and descriptive content analysis was used to identify and synthesize the key themes from the open-ended questions. Ethics approval was obtained from Hamilton Integrated Research Ethics Board (Project# 2017-0977).

## Results

### Google Analytic Data (Tracking the Reach)

**Figure 1:**
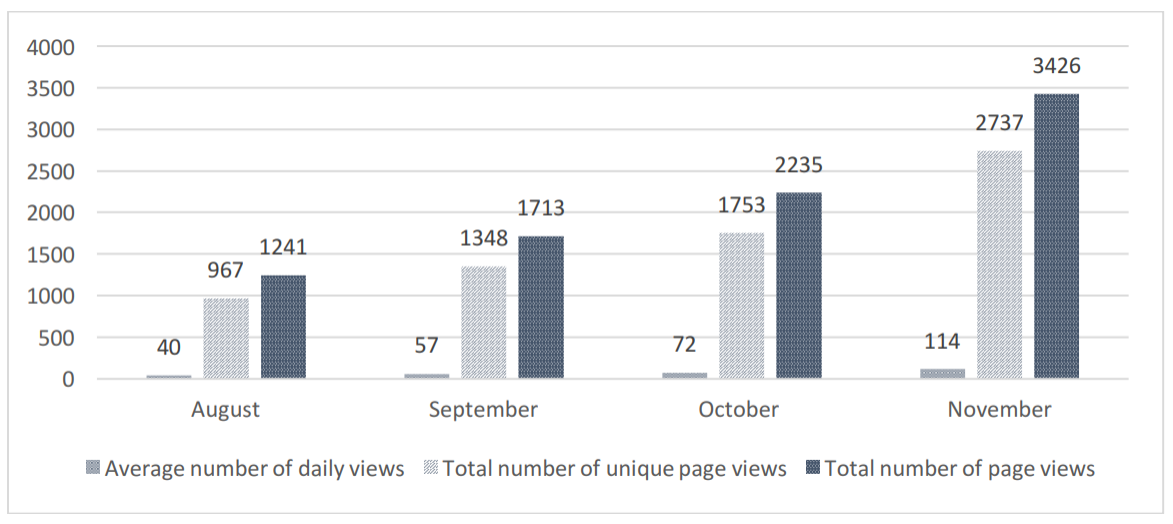
Number of visits to the Knowledge Hub.

Over the four-month evaluation period (August-November 2017), there were over 6,800 unique visitors to the Knowledge Hub, with the number of visitors increasing each month (Figure 1). This could correspond with KT strategies implemented by the research team (eg, conference presentations, educational outreach visits, monthly *CanChild* newsletters featuring the Knowledge Hub) and spread of the Knowledge Hub by people who liked and were sharing it within their communication channels and social networks.

### Survey Responses

### Survey Completion

The survey went live on November 3, 2017, and data were collected for 1 month. A total of 87 respondents provided information, with 63 completing the full survey and 24 partially completing the survey (ie, 1 or 2 sections), providing a completion rate of 72%. Most people visited the Knowledge Hub 1-5 times (n=63) and spent up to 45 minutes exploring the hub (n=61) prior to providing feedback. The following results were based on the survey data.

#### Respondent Demographics

Just under half the respondents that completed the survey lived in Canada (42/87, 48%). The only other country with >10 respondents was the United States (17/87, 20%). The remainder of respondents came from 13 countries. Respondents were asked to state the perspective from which they were viewing the Knowledge Hub (eg, family member, clinician etc). Of the 87 people who completed the survey, 42 were clinicians and 30 were family members (n=20) or individuals with a disability (n=10). There was a wide distribution of perspectives with many respondents (n=36) falling into >1 stakeholder category (Table 1).

#### Respondents’ Familiarity with the “F-words”

The majority of people (62/87, 71%) had heard of the “F-words” prior to visiting the Knowledge Hub and either “extremely liked the ideas” (38/62, 61%) or “liked the ideas” (21/62, 34%). Of the 62 people who were familiar with the “F-words”, 43 (69%) felt confident identifying and explaining the “F-words” ideas, 37 (60%) had shared them with others, and 35 (56%) indicated that they had used or applied them in practice prior to exploring the hub. To understand how people were using or applying the “F-words,” we asked for open-ended feedback. The majority of people who provided written responses were clinicians, researchers, people with disabilities, or family members. Depending on the stakeholder group, the use of the “F-words” concepts varied. Examples of how the “F-words” concepts have been used by each stakeholder group are shown in Table 2.

#### Perceived Usefulness of the Knowledge Hub

To evaluate the usefulness of the Knowledge Hub, respondents were asked to rate their overall satisfaction. Of the 87 people who started the survey, 66 people completed this section. Therefore, the following data are based on these 66 responses. Overall, 86% (57/66) of respondents felt the purpose was clear, 92% (61/66) found the Knowledge Hub user-friendly, and 92% (61/66) and 94% (62/66) perceived the content to be meaningful and relevant for families and service providers, respectively. The average scores ranged from 4.23 to 4.39 out of 5 for each category (Table 3).

**Table 1:** Demographic characteristics of respondents (N=87).

Characteristic		Value, n (%)
**Country of residence^a^**		
	Canada	42 (48)
	United States	17 (20)
	Australia	4 (5)
	Spain	3 (3)
	Brazil	3 (3)
	United Kingdom	2 (2)
	Ethiopia	2 (2)
	South Africa	2 (2)
	No answer	5 (6)
**Type of stakeholder^**b,c**^**		
	Clinician^d^	42 (48)
	Researcher	24 (28)
	Family member^d^	20 (23)
	Educator	17 (20)
	Friend of someone with a disability	17 (20)
	Student	13 (15)
	Person with a disability^d^	10 (11)
	No answer	2 (2)
**Gender**		
	Female	69 (79)
	Male	13 (15)
	No answer	5 (6)
**Previously aware of the “F-words”**		
	Yes	62 (71)
	No	19 (22)
	No answer	6 (7)

^a^Includes countries with >1 respondent.

^b^Includes stakeholder groups with >5 respondents.

^c^Some respondents fit into >1 group (eg, clinician and educator).

^d^Primary target audience.

**Table 2:** Examples of use of the “F-words” concepts prior to exploring the hub.

Level of uptake		Sample quotes
**Family**		
	Applied the F-words to their own lives (n=5)	"The F-words are very applicable to my own life. I’m happy to share them with others I feel could also benefit from this framework." [Person with a disability, Canada]
	Used the F-words when speaking with therapists and teachers to assist with goal-setting and planning for their child (n=2)	"When speaking to therapists and teachers in relation to goals for my child." [Family member, Canada]
**Clinical**		
	Implemented the F-words to help with goal-setting with families, to frame conversations with families, and to help guide program planning and decision making (n=15)	"When discussing outcomes and goal planning with the family, we discussed the ICF model and used the F-words as descriptors for the various categories." [Clinician, USA] "Through discussion with families and creating goals that fit families’ lives." [Clinician-researcher, Canada]
**Research or education**		
	Incorporated the F-words into training for students (n=3)	"Especially in educational settings, such as the training of graduates in physiotherapy, multiprofessional residence in children's health, as well as the master's degree in collective health and PhD on rehabilitation sciences." [Clinician-researcher/Educator, Brazil]
	Incorporated the F-words into publications and grant writing applications (n=1)	"Used in talks to families and professional groups. Used in publications and in grant applications." [Clinician-researcher, Australia]
**Health care organization**		
	The F-words are influencing organizations in items such as facility planning, departmental missions, and the development of programs (n=2)	"Facility planning, restructuring." [Administrator, USA] "Used them to guide collaborative goal-setting with families/clients; to focus our departmental mission; to develop programs." [Clinician-researcher/Educator, USA]

**Table 3:** Overall satisfaction with the Knowledge Hub (n=66).

Item	Strongly agree, n (%)	Agree, n (%)	Neutral, n (%)	Disagree, n (%)	Strongly disagree, n (%)	No answer, n (%)
The purpose is clear	32 (48)	25 (38)	5 (8)	4 (6)	0 (0)	0 (0)
The hub is user-friendly	19 (29)	42 (64)	2 (3)	1 (2)	0 (0)	2 (3)
I enjoyed exploring the Knowledge Hub	26 (39)	35 (53)	3 (5)	0	0 (0)	2 (3)
The content is meaningful and relevant for families	32 (48)	29 (44)	4 (6)	1 (2)	0 (0)	0 (0)
The content is meaningful and relevant for service providers	31 (47)	31 (47)	3 (5)	1 (2)	0 (0)	0 (0)

Respondents were also asked to indicate which sections of the Knowledge Hub they liked and what could be improved. A total of 65 people answered this question, all of whom indicated they liked at least one section of the Knowledge Hub, 57% (37/65) indicated that they liked all sections and 45% (29/65) indicated they had no further suggestions for improvements. Table 4 shows the breakdown of the items respondents liked and the possible areas for improvement.

The survey also collected open-ended feedback to gain a better understanding of what were perceived to be the best features of the Knowledge Hub (48 respondents) and which areas needed improvement (25 respondents). The best features and areas for improvement were categorized into two aspects: content and format or design of the Knowledge Hub. The key themes within these areas were then identified based on the number of responses. Table 5 shows the reported best features and Table 6 summarizes the reported main areas for improvement.

**Table 4:** The breakdown of what people liked and what can be improved (n=65).

Item^a^	Liked, n (%)	Areas for improvement, n (%)
Homepage	26 (40)	6 (9)
International Classification of Functioning, Disability and Health Resources	12 (18)	8 (12)
F-words Footprint	14 (22)	2 (3)
Family and Clinician Voices	17 (26)	4 (6)
F-words Tools	23 (35)	7 (11)
Research Team	10 (15)	3 (5)
All of the above	37 (57)	1 (2)
None of the above	0 (0)	29 (45)
Other	1 (2)	9 (14)

^a^People could select >1 item.

**Table 5:** Open-ended feedback on the best features of the Knowledge Hub.

Category		Sample quotes	
**Content**			
	Overall, the videos (n=21) and “F-words” tools (n=15) were identified as the best features of the Knowledge Hub.		"My favorite part of the Hub is the F-words Tools section! As an educator, access to tools and examples from children helps me to understand how the F-words come into practice in the classroom and at home." [Educator, Canada]
	Many people also valued the stories and examples shared by families and clinicians on what the “F-words” meant to them and how they are using the “F-words” in practice (n=9).		"The writing by families and therapists were also quite valuable in seeing how these principles are applied in many different situations. They are also very engaging to read." [Researcher, USA]
**Format or design**			
	Key design features were that the hub was easy to navigate (n=8), user-friendly (n=7), and interesting or engaging (n=7).		"It's simple to use and navigate, visually interesting and love the video content." [Family member, friend, researcher, Canada]
	The hub being publically available with sharable, downloadable content (n=5).		"Sharing the information is great but also providing the tools and resources for families and providers alike is crucial to getting the word out and to helping these families." [Clinician, USA]

**Table 6:** Open-ended feedback on areas for improvement of the Knowledge Hub.

Category		Sample quotes	
**Content**			
	More examples of the application of the F-words and the impact. This includes more case vignettes, as well as formal research studies implementing and evaluating the F-words tools (n=7).		"I think it would be important to expand the dissemination of the six F-words by conducting studies on its application and results obtained." [Clinician-researcher, Brazil]
	Also, extending the F-words to other populations, including teachers, young children, and increasing the diversity of representation (n=4).		"Improve the representation of diverse (SES, racial, ethnic, disabilities) families and practitioners to discuss barriers and different strategies possible in a wide lens." [Person with a disability, family member, friend, student, researcher, educator, USA]
**Format or design**			
	Overall organization (eg, clearly identifying the different sections, resources, purpose of the hub, etc) (n=8).		"The content is excellent, some of the formatting could be improved to make it more user-friendly (lots of scrolling currently and hard to orient to all the great materials with that format)." [Student, researcher, support worker, Canada]
	The need for better navigation from the homepage (n=5).		"Better navigation. From the home page I would like a “how to use this site” section that will guide me to what I need to be looking at use - either as a parent, as a therapist, as a researcher." [Family member, Canada]

**Table 7:** Reported use of the Knowledge Hub (n=63).

The Knowledge Hub...	Strongly agree, n (%)	Agree, n (%)	Neutral, n (%)	Disagree, n (%)	Strongly disagree, n (%)	No answer, n (%)
...increased my understanding of the F-words concepts.	27 (43)	31 (49)	2 (3)	1 (2)	0 (0)	2 (3)
...influenced what I think about the F-words concepts.	20 (32)	29 (46)	11 (17)	1 (2)	0 (0)	2 (3)
...will be useful to me.	23 (37)	32 (51)	7 (11)	0 (0)	0 (0)	1 (2)
...will influence the things I do when I am working with others.	22 (35)	30 (48)	5 (8)	2 (3)	1 (2)	3 (5)

#### Reported Use

The final section of the survey explored the use or intended use of the Knowledge Hub and the “F-words” concepts. Among the people who started the survey, 72% (63/87) completed this final section. The following data are based on responses from these 63 people (Table 7).

Overall, 97% (61/63) people indicated that they either “extremely liked” (42/63, 67%) or “liked” (19/63, 30%) the “F-words” concepts, 92% (58/63) people reported that the hub increased their understanding of the “F-words”, and 78% (49/63) people reported that the hub influenced their thinking about the “F-words”. We were also interested in participants’ confidence in identifying and explaining the F-words after exploring the Knowledge Hub. Overall, 90% (57/63) people indicated that they were either “extremely confident” (19/63, 30%) or “confident” (38/63, 60%). When asked whether the Knowledge Hub would be useful to them, 83% (52/63) people reported that it would influence the way they did things when working with others.

Lastly, respondents were asked to rate the Knowledge Hub as a KT tool for sharing information with families and service providers. Overall, 90% (57/63) people rated it 4 or 5 (on the 5-point Likert scale) as a KT tool for families, 98% (60/63) people rated it as 4 or 5 as a KT tool for service providers, and 97% (58/63) people planned to share the Knowledge Hub.

## Discussion

### Reflections on the Development Process

From the beginning, it was important to us that the Knowledge Hub be cocreated with stakeholders. While our integrated team of families and researchers led the development process, many stakeholders outside of our research team were involved. For example, we worked with clinicians and health care administrators, who we knew were applying the “F-words” to share examples of how they were using the “F-words” in their organizations. These examples then served as examples of application for other service providers.

We believe early stakeholder involvement was crucial not only to the development of a meaningful and relevant resource but also to the dissemination of knowledge regarding the Knowledge Hub. Individuals interested in the Web-based hub were more likely to share it with their own communities, thus increasing its reach and potential impact as it was spread through broad communication channels and social networks [24]. The importance of involving stakeholders such as families and service providers in the development of the KT resources has been recognized by other children’s health researchers [25-27].

Another key feature of the Knowledge Hub was its promotion of knowledge sharing and exchange [28]. In comparison with other Web-based KT resources such as Web-based learning modules which are difficult to change after completion, as the Knowledge Hub is organic, it can be easily adapted and can grow over time. This not only encourages people to return to the Knowledge Hub but also inspires them to get involved and contribute to the conversation (ie, become “knowledge brokers” of the “F-words”) [4,29]. Having the Knowledge Hub freely available is crucial to supporting this global dissemination and uptake.

One common barrier reported in the literature was the time and resources needed to develop and implement KT interventions [3,25,30]. While our research team was responsible for developing and collating the content for the Knowledge Hub, we leveraged many of *CanChild’s* resources (eg, the *CanChild* website and *CanChild* KT staffs’ or students’ time) to design and maintain the Knowledge Hub. Creating and collating content for the Knowledge Hub also took a lot more time than initially expected. The development process involved iterative rounds of feedback from various stakeholders. We did not follow a structured system or timeline for collecting feedback, which led to a longer process. In the future, we would recommend the use of a structured process tailored to collecting feedback from a diverse group of stakeholders [7].

A key facilitator for this project was the use of theory and best practice guidelines to inform the KT intervention [18,31]. The KTA framework [15], the Diffusion of Innovation theory [24], and Levac et al’s [7] best practice guidelines for developing Web-based educational resources were all used to inform the development process. Specifically, the KTA framework [15] provided the “big picture” and was used as the overarching guide for the KT process. Levac et al’s [7] best practice guidelines for Web-based KT resources helped us with specific details and steps needed to design the Knowledge Hub. These guidelines were useful as they were specifically tailored to our chosen KT strategy. Lastly, the Diffusion of Innovation theory informed the design and implementation of the Knowledge Hub through consideration of the characteristics of the innovation that support adoption (ie, relative advantage, complexity, compatibility, trialability, and observability), as well as the key factors that influence innovation dissemination (ie, time, social networks, and communication channels) [24].

### Evaluation Reflections

The main aim of the Knowledge Hub is to inform families and service providers about the “F-words”/ICF concepts and to provide action-oriented tools to support the uptake and use of the “F-words” in practice. As such, the goal of this pilot evaluation was to evaluate the usability and utility of the Knowledge Hub. The findings from this study revealed that these self-assigned goals were attained. Overall, the respondents reported that the Knowledge Hub was informative and useful and the “F-words” tools were one of the best features of the Knowledge Hub.

In general, the hub received high ratings with regard to both its *perceived usefulness* and *potential use*. While mixed-model analyses between groups were not completed, the high ratings given by all participants implied that the Knowledge Hub was perceived to be a meaningful resource for both service providers and families. This finding was consistent with earlier research from *CanChild* that found that when educational materials were clearly written and user-friendly, they were useful and impactful for multiple target audiences (ie, families and service providers) [32,33]. Furthermore, while more structured research is still needed to evaluate the impact of the Knowledge Hub on family and service provider behavior, people’s reported intentions to use the hub were an encouraging preliminary finding. As outlined in behavior change literature, people’s attitudes have a significant influence on whether a change will happen [34,35].

We recognize that prior to exploring the Knowledge Hub, over 70% of people who completed the survey had previously heard of the “F-words.” Of these respondents, the majority felt confident identifying and explaining the “F-words” ideas, and about half of them indicated that they had used or applied the “F-words” in practice. Despite many respondents already being familiar with the “F-words” concepts, the majority stated that the Knowledge Hub increased their understanding of the “F-words” ideas. This is an important finding as it implies that the Knowledge Hub can increase perceived knowledge even if individuals have prior familiarity with the concepts. This probably occurred because the resources provide tangible materials that move beyond simple concept familiarity. Unfortunately, due to the low response rate from people for whom the “F-words” concept was new, it is not possible to say whether the Knowledge Hub is useful across all adopter categories (ie, from the *early adopters—* those who are already using the “F-words”—to the *late adopters—* those to whom the “F-words” are new) [24].

Conducting a pilot evaluation of the usability and utility of the Knowledge Hub is an important step toward ensuring its overall impact and sustainability [7,23]. This pilot evaluation helped us to understand what people liked about the Knowledge Hub (eg, the videos, “F-words” tools, families’ and clinicians’ voices, etc) and what changes were needed to improve it (eg, re-organizing the homepage to support navigation throughout the hub). The evaluation also helped us understand who was accessing the Knowledge Hub (ie, mostly the *early adopters* of the “F-words” concepts) and what was needed to broaden the applicability of the Knowledge Hub to a wider audience (eg, extending the “F-words” to other populations and conducting research on the impact of using the “F-words” tools). These findings will both inform and complement future evaluations of the Knowledge Hub. Recognizing that experimental evaluations only identify whether an intervention is effective, process evaluations such as this are recommended to understand the reasons why interventions are (or are not) effective [36,37].

### Study Limitations and Future Directions

Based on the respondents’ positive feedback, we anticipate that the Knowledge Hub will be a useful resource for both families and service providers. A limitation to this work is that feedback was gained from only a small sample of the people who visited the hub during this period. It is important to remember that the majority of people who provided feedback were those who were already familiar with the “F-words” concepts and also liked the “F-words” ideas. Thus, their potential biases must be recognized.

In order to reach a broader audience, more time is needed to actively disseminate the Knowledge Hub. While the preliminary findings after a one-month evaluation were reported here, in order to overcome selection bias (ie, those who already like the F-words ideas), the evaluation will remain posted on the Knowledge Hub and further feedback will be monitored. The hope is that over time more people (including those who are not already familiar with the “F-words”) will complete the survey.

The next step is to evaluate the impact of the Knowledge Hub and “F-words” concepts at the family, clinician, and organizational levels. As active implementation strategies are useful in supporting the dissemination and uptake of educational materials, we plan to combine the Knowledge Hub intervention with tailored invitational outreach visits to local children’s treatment centers (CTCs). Once again, this is a stakeholder-driven strategy as CTCs have contacted us and expressed a need for in-person educational training on the “F-words” concepts. Based on our positive experiences of working with families and service providers to develop the Knowledge Hub, this project will continue to be informed by an iKT strategy.

### Conclusions

Working with families and service providers, we designed a theory-informed and evidence-informed Web-based KT resource that was perceived to be relevant and meaningful to families raising children with disabilities and to service providers working in the field. To date, the Knowledge Hub has mainly reached *early adopters* (ie, people who like the “F-words” ideas and are seeking more information) [24]; therefore, to reach a wider audience (ie, the *early majority*), further active implementation strategies are needed.

KT is not only the doing but also the studying of the KT process and outcomes. From the evaluation of the usability and utility of the Knowledge Hub, we now have a good understanding of what was done well and what can be improved. Based on the findings from this pilot study, we intend to make minor changes to the Knowledge Hub before conducting a larger evaluation study of the impact at the family, clinician, and organizational levels. Knowledge gained from this study is transferrable to other KT initiatives involving families and service providers. We hope that the findings and lessons learned from this integrated KT project will assist others in advancing iKT science and practice in other areas of childhood disability research.

## Appendix

Multimedia Appendix 1 Application of Levac et al’s (2015) recommended best practices of Web-based knowledge translation resources.

Multimedia Appendix 2 Description of “F-words” Knowledge Hub.

Multimedia Appendix 3 “F-words” Knowledge Hub evaluation recruitment poster.

## Abbreviations

CTC: children’s treatment center
ICF: International Classification of Functioning, Disability and Health
iKT: integrated knowledge translation
KT: knowledge translation
KTA: Knowledge-to-Action
