# Supplementary material for: A Web-Based Knowledge Translation Resource for Families and Service Providers (The “F-Words” in Childhood Disability Knowledge Hub): Developmental and Pilot Evaluation Study
Source: JMIR Rehabil Assist Technol. 2018 Dec 21;5(2):e10439. doi: 10.2196/10439 (PMC6331144; doi:10.2196/10439)
Supplement: Multimedia Appendix 1 [file rehab_v5i2e10439_app1.pdf]

Recommendations	Steps	Application in the Current Study
1. **Develop evidence-based, user-centered content**	1. Assess audience needs	• Informal needs assessment through 'F-words' awareness video survey and consultation with families and service providers<br>• Scoping review conducted to explore KT strategies targeting family stakeholders
	2. Summarize evidence to address audience needs	• Research team brainstormed information and sections to include in online resource<br>• Key messages re: 'F-words' & ICF concepts identified for families + service providers<br>• Categorized information based on 'need to know' vs. 'nice to know'<br>• 'Need to know' (text embedded in website) vs. 'Nice to know' (links provided to papers and other resources for more information)
	3. Use theory, framework, or model	• KTA framework informed the development process<br>• The Diffusion of Innovation theory characteristics of innovation considered when designing the resource
	4. Select an appropriate KT format	• Online Knowledge Hub hosted on *CanChild* website<br>• Incorporated content to meet the needs of different learning styles (e.g., included written information, videos, podcasts, downloadable fillable tools, etc.)<br>• Included information created by people outside of the research team to build connections and an environment for knowledge sharing and exchange
	5. Develop learning objectives	• Identified goals and learning objectives for Knowledge Hub<br>• Developed a purpose statement
	6. Include multimedia content	• Videos, webinars, podcasts, presentation recordings posted on Knowledge Hub
2. **Tailor content to online**	1. Partner with a web	• Worked with *CanChild's* media and website specialists and students

**format**	developer	
	2. "Mock up" content and navigational structure	• Used PowerPoint to mock up website and then transferred content to website<br>• Mapped out each section including navigational structure
	3. Consider web sustainability	• Goal for website to be a 'living' document that can be continually updated<br>• Designated internal *CanChild* staff member and KT students assist with keeping the website up to date
	4. Pilot test with intended audience	• Informal pilot test with families, service providers, students and researchers affiliated with *CanChild* to pilot website
3. **Evaluate impact**	1. Embed evaluation within resource	• Pilot evaluation (anonymous survey) to evaluate utility (reach, usefulness, and use) of the Knowledge Hub
	2. Collect usage data	• Google analytics used to track number of visits to Knowledge Hub<br>• Further analysis can be run (e.g., number of times people access, avg. time spent per visit, popular resources vs. those overlooked, etc.)
	3. Build in methods to evaluate short-term and long-term learning, retention, and behavior change	• Will be incorporated into follow-up studies to evaluate impact of Knowledge Hub at the family, clinician, and organizational levels<br>• Mixed-methods evaluation including quantitative questionnaires exploring participants' self-reported change in knowledge, attitudes, behaviour and qualitative interviews to gain more in-depth understanding
4. **Share results and disseminate the knowledge**	1. Write scientific papers to report your results	• This article reports on the development process and preliminary findings from the pilot evaluation of the Knowledge Hub.

	2. Share results with participants to disseminate information to your target audience	• An 'In Brief' (lay summary) will be written on findings from the pilot evaluation and posted on the Knowledge Hub<br>• Will also share findings through presentations and webinars<br>• Social media and *CanChild* Today newsletter will share updates
	3. Maintain knowledge "currency"	• KT specialists at *CanChild* will be responsible for keeping Knowledge Hub up to date

3
