# Supplementary material for: A Web-Based Knowledge Translation Resource for Families and Service Providers (The “F-Words” in Childhood Disability Knowledge Hub): Developmental and Pilot Evaluation Study
Source: JMIR Rehabil Assist Technol. 2018 Dec 21;5(2):e10439. doi: 10.2196/10439 (PMC6331144; doi:10.2196/10439)
Supplement: Multimedia Appendix 2 [file rehab_v5i2e10439_app2.pdf]

Section	Description
F-words Homepage	• General introduction to the Knowledge Hub.<br>• Introduction video welcomes people to the hub and provides an overview of the various sections, tools, and resources.<br>• Includes written text that identifies and defines the six 'F-words', provides a brief summary of the ICF framework, and shows both the ICF and 'F-words' frameworks.<br>• Two 'F-words' awareness videos, created by parents and youth with disabilities featured to capture visitors' attention and engage the audience.<br>• Embedded link to the open access 'F-words' publication provided.
ICF Resources	• Written text provides key messages regarding the ICF framework.<br>• Sub-headings include: 1) What is the ICF Framework? 2) Why is it important? and 3) What does it mean for families and service providers?<br>• Links to additional ICF resources for people who are interested in learning more provided.
F-words Footprint	• Highlights how the 'F-words' are being shared and used around the world. People are invited to contribute to this section – foster knowledge sharing and exchange.<br>• Includes a list of the publications, an interactive map highlighting the presentations given around the world, videos on the 'F-words', podcasts, webinars, downloadable PDFs of posters presented on the 'F-words', and links to online blogs and news articles that have featured the 'F-words' ideas.<br>• Features the 'F-words' poster, created in collaboration with World CP Day in 2016, and now translated into < 25 languages.
Family & Clinician Voices	• Features how parents, youth with disabilities and clinicians are talking about and applying the 'F-words' (bring the 'F-words' to life).<br>• The quotes are taken from news articles and online blogs written by people around the world and the pictures have been shared by families and service providers.
F-words Tools	• Section 1: Interactive, downloadable tools created by our research team, including the 'F-words Agreement', 'F-words Photo Collage', 'F-words Goal Sheet', and

	‘F-words Profile’.<br>• Section 2: Real-life examples of how clinicians and organizations have applied the ‘F-words’ in their individual work settings.
F-words Research Team	• Introduces each member of our integrated research team (picture and bio provided).

2
