# Supplementary figures and images for: A Web-Based Knowledge Translation Resource for Families and Service Providers (The “F-Words” in Childhood Disability Knowledge Hub): Developmental and Pilot Evaluation Study
Source: JMIR Rehabil Assist Technol. 2018 Dec 21;5(2):e10439. doi: 10.2196/10439 (PMC6331144; doi:10.2196/10439)

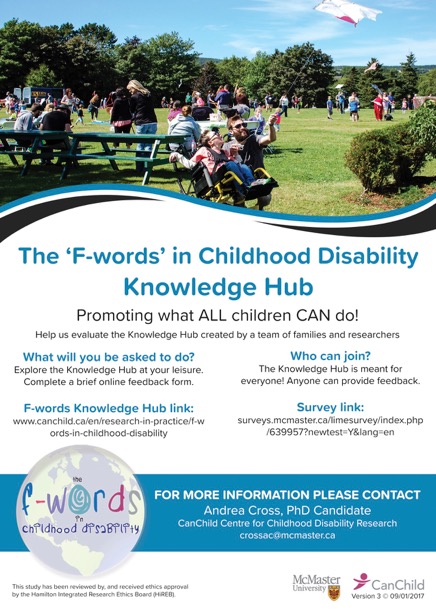

Supplement: Multimedia Appendix 3 [file rehab_v5i2e10439_app3.jpg]
